# Artificial Intelligence for Early Detection and Prediction of Chronic Obstructive Pulmonary Disease Exacerbations

**DOI:** 10.3390/healthcare14060806

**Published:** 2026-03-21

**Authors:** LeAnn Boyce, Victor Prybutok

**Affiliations:** 1 Department of Analytics and Statistics, College of Science, University of North Texas, Denton, TX 76203, USA; 2 Department of Information Technology and Decision Sciences, G. Brint Ryan College of Business, University of North Texas, Denton, TX 76203, USA; victor.prybutok@unt.edu

**Keywords:** chronic obstructive pulmonary disease (COPD), COPD exacerbation, artificial intelligence, machine learning, deep learning, wearable sensors, electronic health records, explainable artificial intelligence, clinical decision support, precision medicine

## Abstract

**Highlights:**

**What are the main findings?**

- Studies consistently show that combining frequent health data with contextual information improves the early detection of COPD exacerbations.
- Explainable Artificial Intelligence (AI) approaches help clarify why risk increases, revealing early warning patterns and meaningful differences in how patients respond to environmental exposures.

**What are the implications of the main findings?**

- For AI tools to be useful in practice, COPD exacerbations must be defined consistently, and models must be validated across diverse patient populations and care settings.
- When designed with clinical workflows in mind, interpretable AI systems could support earlier intervention and more personalized COPD management.

**Abstract:**

Background: Exacerbations of chronic obstructive pulmonary disease (COPD) are a leading cause of morbidity, mortality, and healthcare burden worldwide. Early detection and timely intervention remain important challenges in COPD management, given the unpredictable nature of acute deterioration and limitations of traditional spirometry-based risk assessment. Methods: This narrative review synthesizes artificial intelligence (AI)-driven approaches for predicting and detecting chronic obstructive pulmonary disease (COPD) exacerbations across electronic health records, wearable sensors, imaging, environmental data, and patient-reported outcomes, emphasizing novel discoveries and emerging relationships rather than predictive performance. Results: Three major discoveries have been made. First, measurable physiological and behavioral deterioration may precede symptom recognition by approximately 7–14 days, thereby establishing a potential intervention window for anticipatory care. Second, machine learning (ML) models integrating pollutant exposure, medication adherence, and clinical characteristics have identified phenotypes with differential environmental sensitivity, including unexpected exposure–adherence interactions. Third, deep neural network analysis of full spirometry curves has revealed structural phenotypes beyond traditional Forced Expiratory Volume (FEV_1_)-based measures and novel imaging biomarkers. The predictive performance ranges from the Area Under the Curve (AUC) 0.72–0.95, with a pooled meta-analytic AUC of approximately 0.77. Conclusions: AI has uncovered hidden patterns in the progression of COPD, supporting a shift from reactive to anticipatory management. Translation to routine care requires prospective validation, improved interpretability, workflow integration, and generalizability and equity.

## 1. Introduction

Chronic respiratory diseases represent a major global public health challenge, contributing substantially to morbidity, mortality, and healthcare utilization worldwide. Among these, chronic obstructive pulmonary disease (COPD) is one of the leading causes of death and disability, driven by population aging, persistent exposure to environmental risk factors, and increasing prevalence of comorbid conditions. COPD is characterized by progressive airflow limitation, chronic inflammation, and recurrent episodes of acute clinical deterioration known as exacerbations. These exacerbations are pivotal events in the disease course, accelerating lung function decline, increasing the risk of hospitalization, and contributing significantly to mortality and healthcare burden.

The global economic impact of COPD is profound. It is estimated that by 2050, the direct costs of COPD will reach approximately $24.35 trillion, with indirect costs approaching $15.43 trillion, while exacerbations alone account for an additional $15.60 billion [1]. In the United States, COPD-related healthcare expenditures were approximately $31.3 billion and are projected to increase to $60.5 billion by 2029 [2]. Beyond economic consequences, exacerbations substantially impair quality of life, leading to prolonged recovery periods, reduced physical function, and irreversible disease progression [3].

Early identification and management of exacerbations are critical for improving outcomes. When treatment is initiated within 48 h of symptom onset, interventions such as bronchodilator escalation, antibiotics, or systemic corticosteroids can reduce symptom severity, shorten recovery time, and decrease hospitalization risk by up to 40% [4]. However, exacerbations are inherently complex and multifactorial events. They arise from dynamic interactions between physiological deterioration, environmental exposures (such as air pollution and viral infections), comorbidities, behavioral factors, and medication adherence. Importantly, these processes often begin days to weeks before clinical symptoms become apparent, with subtle changes occurring approximately 7–14 days prior to overt deterioration [5].

Despite this window of opportunity, conventional approaches to exacerbation prediction remain largely reactive. Current clinical strategies rely on historical exacerbation frequency, patient-reported symptoms, and clinician judgment, all of which have limited sensitivity for detecting early deterioration. These methods fail to capture the complex, nonlinear relationships and temporal dynamics underlying COPD progression, highlighting a critical gap in proactive disease management.

Recent advances in digital health and data availability have created new opportunities to address this challenge. The increasing use of electronic health records, wearable devices, environmental monitoring systems, and patient-reported outcome platforms has generated large volumes of heterogeneous, high-dimensional health data. These developments have catalyzed growing interest in analytical approaches capable of extracting clinically meaningful patterns from such complex datasets.

Within this context, artificial intelligence (AI) and machine learning have emerged as promising tools for improving early detection and prediction of COPD exacerbations. Unlike traditional statistical methods, AI models can identify nonlinear relationships, temporal trends, and subtle signals of deterioration that may not be detectable through conventional analysis. Several recent studies illustrate this potential. Wu et al. [6] demonstrated that wearable-derived activity data could predict exacerbations up to 7 days in advance with 92.1% accuracy, revealing declines in physical activity that preceded symptom recognition. Similarly, Atzeni et al. [7] combined personal air quality monitoring with machine learning to achieve AUC values of 0.90 (±0.05), uncovering individualized responses to environmental exposures that are not captured by standard clinical thresholds. More recently, Jeon et al. [8] showed that deep learning applied to spirometry curve imaging could predict moderate-to-severe exacerbations (AUC 0.755–0.713), identifying physiological abnormalities beyond conventional spirometric indices.

These findings suggest that AI-driven approaches have the potential to shift COPD management from a reactive model focused on treating acute events to a proactive strategy centered on early detection and prevention. By integrating diverse data sources, including physiological signals, environmental exposures, clinical records, and behavioral patterns, AI models offer a more comprehensive representation of disease dynamics.

However, despite rapid methodological advances and promising predictive performance, translation of AI-based models into routine clinical practice remains limited. Many studies are conducted in controlled research environments and face challenges related to generalizability across populations, data quality and completeness, interpretability of model outputs, and integration into clinical workflows. Additional barriers include alert fatigue, algorithmic bias, and the lack of prospective validation. A recent systematic review and meta-analysis reported that machine learning models did not significantly outperform traditional severity scores for long-term prognosis prediction (pooled *p* = 0.24) [9], suggesting that the benefits of AI may be context-dependent and more pronounced in short-term prediction where dynamic data provide added value.

Although previous systematic reviews have focused primarily on predictive accuracy and methodological comparisons [9,10], less attention has been given to the broader insights generated by AI-driven analyses. Specifically, an important question remains: beyond prediction, what new understanding has AI provided about the mechanisms and patterns underlying COPD exacerbations? Emerging evidence suggests that AI can reveal previously unrecognized disease trajectories, complex interactions between environmental and physiological factors, and heterogeneity in patient responses that are not captured by traditional analytical approaches.

Given the rapid expansion and fragmentation of the literature, there is a need for a comprehensive, clinically oriented synthesis that not only evaluates predictive performance but also examines the novel insights generated by AI applications. This narrative review aims to address this gap by exploring AI-based approaches for early detection and prediction of COPD exacerbations, with a focus on emerging methodologies, integration of diverse data sources, newly identified disease patterns, reported performance across studies, implementation challenges, and future directions for clinical translation.

By critically evaluating both the potential and limitations of AI-driven approaches, this review seeks to inform the development of clinically meaningful, trustworthy, and implementable tools that can improve the management of COPD exacerbations.

## 2. Materials and Methods

This narrative review was conducted using a structured and transparent search strategy to synthesize current evidence on artificial intelligence-based prediction of COPD exacerbations. Although this study was not designed as a formal systematic review or meta-analysis, methodological rigor was applied to minimize selection bias and enhance reproducibility.

### 2.1. Search Strategy

We searched electronic databases, including PubMed, Scopus, IEEE Xplore, and Web of Science, for studies published between January 2015 and January 2026. Search terms included combinations of “chronic obstructive pulmonary disease,” “COPD,” “acute exacerbation,” “artificial intelligence,” “machine learning,” “deep learning,” “predictive modeling,” “readmission,” “remote monitoring,” and “digital inhaler.”” Boolean operators (AND/OR) were used to refine the search strategy.

### 2.2. Eligibility Criteria and Study Selection

In light of the rapidly expanding and methodologically heterogeneous literature on AI applications in COPD, defined inclusion and exclusion criteria were implemented to ensure conceptual coherence while maintaining breadth across data sources and modeling approaches. Eligible records comprised original research studies published in English that developed or validated artificial intelligence (AI) or machine learning (ML) models for (1) COPD exacerbation prediction, (2) hospital readmission prediction, or (3) ICU admission or mortality prediction in adult COPD populations. Studies using EHRs, wearable sensors, imaging, environmental data, or patient-reported outcomes were considered.

Excluded records included studies focused on pediatric populations, non-AI statistical-only analyses, case reports, editorials, and conference abstracts lacking original data.

Following the removal of duplicates, authors screened titles and abstracts to assess relevance to AI-driven predictive modeling in COPD. Full-text manuscripts were reviewed when abstracts provided insufficient detail. Studies were included if they reported predictive performance metrics (e.g., AUC, sensitivity, specificity) or described model architecture and data inputs relevant to exacerbation or related outcomes. Through independent and unanimous agreement, authors finalized a dataset comprising 55 peer-reviewed articles, six papers from proceedings, and one book.

The addition of six conference proceedings papers and one book were included to provide methodological or clinical context relevant to emerging artificial intelligence applications in COPD. These sources were not used as primary evidence for model performance or predictive accuracy but were incorporated where they provided foundational descriptions of emerging technologies, conceptual frameworks, or clinical background necessary to interpret the peer-reviewed literature. All quantitative findings and conclusions regarding predictive model performance are derived exclusively from peer-reviewed studies.

**Figure 1:**
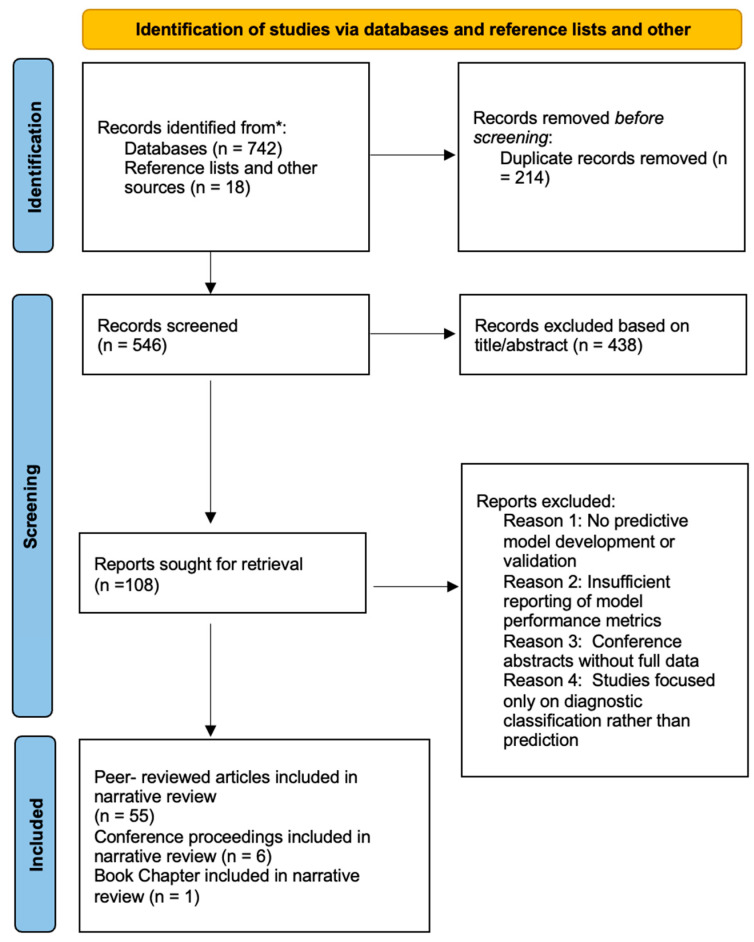
PRISMA-informed flow diagram illustrating the literature identification, screening, eligibility assessment, and final inclusion process used in this narrative review. * Because the included studies exhibited substantial heterogeneity in artificial intelligence methodologies, outcome definitions, and patient populations, the review was conducted as a structured narrative synthesis rather than a formal systematic review or meta-analysis.

Figure 1 illustrates a PRISMA-style flow diagram illustrating the study identification, screening, eligibility assessment, and inclusion process. A total of 760 records were initially identified through database searches and reference screening. After duplicate removal and title/abstract screening, 108 articles underwent full-text review. Sixty-two sources met the inclusion criteria and were incorporated into the narrative synthesis.

The study selection process was documented using a PRISMA-informed flow diagram to improve transparency in reporting the identification, screening, and eligibility assessment of the literature. Because the included studies exhibited substantial heterogeneity in artificial intelligence methodologies, outcome definitions, and patient populations, the review was conducted as a structured narrative synthesis rather than a formal systematic review or meta-analysis.

### 2.3. Data Extraction and Thematic Synthesis

Data extracted from eligible studies included model type (e.g., random forest, gradient boosting, neural network), data sources (e.g., electronic health records, imaging, wearable sensors, and environmental data), outcome definitions, performance metrics, and validation strategies. To facilitate synthesis, the studies were grouped into five thematic domains: (1) EHR-based prediction, (2) imaging and spirometry-based modeling, (3) wearable and digital inhaler monitoring, (4) environmental exposure modeling, and (5) multimodal integration approaches.

### 2.4. Assessment of the Methodological Quality

Given the methodological heterogeneity across the included studies, including variations in data sources, modeling frameworks, outcome definitions, and validation strategies, formal quantitative bias scoring was not performed. A structured qualitative appraisal was conducted to evaluate methodological robustness and translational relevance. Sample size adequacy and event-per-variable ratios; the presence or absence of external validation cohorts; safeguards against overfitting, including cross-validation or regularization techniques; transparency and completeness of reporting regarding model architecture and feature selection; and the extent to which findings could be generalized across healthcare systems, geographic regions, and patient populations were all examined. This approach allowed a comparative evaluation of evidentiary strength while acknowledging the evolving nature of research on COPD driven by AI.

### 2.5. Use of Generative Artificial Intelligence

**Figure 2:**
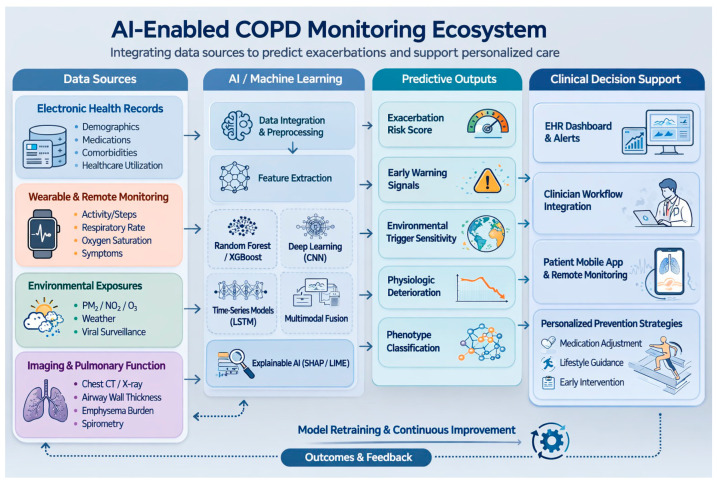
Conceptual architecture of an AI-enabled COPD monitoring ecosystem. The conceptual illustration was generated with assistance from ChatGPT 5.2 Instant and subsequently reviewed and edited by the authors.

During manuscript preparation, generative artificial intelligence tools (Claude Sonnet 4.6 and ChatGPT 5.2) were used to assist with language editing, formatting, and the generation of a conceptual illustration (Figure 2). The AI-generated content was reviewed, revised, and validated by the authors to ensure accuracy and alignment with the scientific literature. No AI tools were used for data analysis, data interpretation, or generation of scientific conclusions. The authors take full responsibility for the integrity and content of the manuscript.

## 3. Results

The following sections synthesize findings across thematic domains rather than presenting individual studies sequentially. Emphasis is placed on identifying methodological patterns, comparative strengths and weaknesses of different AI architectures, and recurring study design limitations that influence predictive performance and clinical applicability. Representative study characteristics and selected performance metrics are summarized in Table 1, Table 2 and Table 3 to support cross-study comparison.

### 3.1. AI-Enabled Discoveries in the Prediction of COPD Exacerbation

Artificial intelligence (AI) methods identify patterns and predictive signals within complex datasets by modeling nonlinear interactions among clinical, physiological, behavioral, and environmental variables that are difficult to capture using traditional regression-based approaches. Unlike conventional statistical models that rely on predefined relationships and linear assumptions, AI-driven methods can integrate heterogeneous data sources and adapt to complex disease dynamics. These approaches have improved risk stratification and revealed new insights related to temporal destabilization, environmental sensitivity, and structural phenotyping that extend beyond traditional COPD assessment frameworks [37,38].

#### 3.1.1. Discovery of Preclinical Physiological Deterioration

Short- to near-term predictive models, ranging from daily monitoring to 7-day windows, prioritize early warning and rapid intervention by capturing dynamic clinical status and environmental risk factors. Wu et al. [6] demonstrated strong performance for 7-day exacerbation prediction using wearable and environmental data, while Jo et al. [11] developed a daily risk prediction model integrating clinical history with weather conditions, air pollution, and respiratory virus surveillance. At even shorter horizons, Chmiel et al. [12] used patient-reported data from a digital health application to support 3-day prediction. Although discrimination was moderate, threshold tuning enabled high-sensitivity early alerting systems capable of supporting preemptive interventions such as medication adjustment or intensified monitoring (Table 1).

Deep learning approaches have gained attention because they can process high-dimensional time-series and image data without extensive manual feature engineering. Wu et al. [6] showed that wearable-derived activity measures and environmental exposures can support accurate short-term AECOPD prediction. Similarly, Jeon et al. [8] demonstrated that transformer-based analysis of spirometry flow-volume and volume-time curves could predict moderate-to-severe exacerbations within one year, outperforming clinical-only models. These findings suggest that subtle physiological signals embedded in continuous monitoring data or pulmonary function curves may precede clinically detectable deterioration (Table 1 and Table 2).

Hybrid temporal approaches integrating multiple prediction windows may further improve clinical usefulness by combining long-term risk stratification with short-term alerts. Although most current models operate within a single prediction horizon, integrating complementary temporal perspectives may better align predictive modeling with real-world clinical workflows.

#### 3.1.2. Discovery of Environmental Sensitivity and Contextual Triggers

Most AI applications in COPD exacerbation prediction rely on supervised machine learning models trained on labeled datasets to identify patterns associated with future exacerbation risk [35,39]. Common approaches include decision trees, random forests, support vector machines, and gradient boosting algorithms such as XGBoost, LightGBM, and CatBoost.

Random forest models have shown strong performance when integrating clinical and environmental variables. Atzeni et al. [7] used personal air quality monitoring data combined with clinical information to identify COPD subgroups with distinct environmental sensitivities. Feature attribution analyses revealed that pollutant exposure (PM_2.5_ and NO_2_) influenced exacerbation risk differently across patient subgroups, highlighting heterogeneity that is often obscured in population-level analyses. Liao et al. [13] similarly reported strong random forest performance when predicting exacerbations 3 and 6 months in advance using electronic medical record data (Table 1).

Gradient boosting methods have also demonstrated strong predictive performance due to their ability to capture nonlinear relationships, handle heterogeneous data types, and manage missingness with minimal preprocessing. Jo et al. [11] used LightGBM to integrate clinical variables with meteorological conditions, air pollution, and viral surveillance data, demonstrating that environmental and viral indicators contribute meaningfully alongside prior exacerbation history. Collectively, these studies suggest that COPD exacerbation risk is influenced by dynamic environmental and contextual factors rather than static clinical variables alone (Table 1 and Table 2).

#### 3.1.3. Discovery of Healthcare Use and Predictors of Medication Use

Longitudinal healthcare data provide additional predictive signals for exacerbation risk. Zeng et al. [14] developed a large-scale gradient boosting model using electronic health record data to predict severe exacerbations requiring emergency department visits or hospitalization. The study demonstrated that medication possession ratios and recent healthcare utilization patterns were more predictive than prescribed medication dosages or traditional comorbidity indices. Selected laboratory markers, including eosinophil counts and C-reactive protein, also contributed incremental predictive value beyond conventional clinical assessments (Table 1 and Table 2).

Support vector machines have shown utility in settings with limited sample sizes and reduced feature sets. Kor et al. [15] reported performance comparable to gradient boosting approaches when predicting first-time COPD exacerbations using registry data and recursive feature elimination to reduce dimensionality. More broadly, several studies have focused on long-term risk stratification over windows ranging from 3 months to 1 year, enabling identification of patients who may benefit from preventive interventions and care management strategies rather than immediate clinical response [40,41].

#### 3.1.4. Discovery of Structural Phenotypes Beyond Traditional Indices

Deep learning methods have also expanded the use of imaging data in COPD research. González et al. [42] demonstrated that deep learning analysis of chest computed tomography (CT) images captures structural lung abnormalities associated with disease severity and prognosis beyond traditional quantitative imaging metrics. Zhu et al. [16] further showed that integrating self-supervised deep learning features with radiomics and epidemiological questionnaire data improved diagnostic discrimination compared with either approach alone (Table 2).

Unsupervised learning techniques provide an additional perspective by identifying latent disease patterns without requiring labeled outcomes. Atzeni et al. [7] used clustering methods to identify COPD phenotypes with distinct environmental response patterns, while Almeida et al. [17] applied anomaly detection to chest CT imaging to differentiate low-risk individuals from COPD patients. These approaches highlight the potential of AI to reveal structural and subgroup phenotypes that are not captured by conventional spirometric or imaging summaries.

#### 3.1.5. Enabling AI Methods and Emerging Approaches

Traditional machine learning methods and deep learning architectures differ not only in complexity but also in the types of data they are best suited to analyze. Comparative studies suggest that ensemble methods such as gradient boosting remain among the most robust approaches for COPD prediction tasks involving high-dimensional structured data. For example, Jang et al. [39] reported that XGBoost outperformed several other ensemble methods when applied to clinical and sleep-derived COPD datasets (Table 2).

Emerging AI methods may further expand the COPD prediction landscape. Variational autoencoders have demonstrated promise for unsupervised imaging feature extraction and multimodal integration [16]. Graph neural networks can model inter-patient similarity structures and capture disease heterogeneity in radiomics-based COPD classification [43]. Federated learning may enable privacy-preserving multi-site model development using distributed datasets [31,32,33,34], while transfer learning and few-shot learning may improve prediction in settings where labeled COPD data are limited [44,45,46,47,48]. Transformer-based architectures and attention mechanisms are also increasingly used for modeling longitudinal clinical data because they capture long-range temporal dependencies and improve interpretability [23,43,49,50]. Hybrid CNN–LSTM architectures represent another promising direction for multimodal time-series modeling using wearable and environmental sensor data [10,51,52].

#### 3.1.6. Explainable Artificial Intelligence, Clinical Utility, and Workflow Integration

A major challenge in COPD AI research is the black-box nature of complex machine learning models, which can achieve high predictive accuracy while offering limited insight into the rationale underlying predictions [15,41]. Explainable artificial intelligence (XAI) approaches aim to address this limitation by providing interpretable explanations of model behavior.

Among these approaches, SHAP has become the most widely used interpretability method in COPD prediction studies, providing both global feature importance and patient-specific explanations [7,15,41]. LIME offers a complementary approach by approximating model behavior near individual predictions using simpler surrogate models [24,25]. Attention visualization and gradient-based techniques further support interpretability by highlighting spatial regions or temporal patterns that contribute to predictions in image-based and longitudinal models [23,53,54].

Clinical utility extends beyond interpretability. Decision curve analysis and net benefit methods evaluate whether prediction models would improve outcomes across clinically relevant intervention thresholds [55,56]. Increasingly, studies report these metrics alongside discrimination measures, reflecting growing emphasis on real-world impact (Table 3) [57,58].

Successful integration into clinical workflows requires that AI systems augment rather than replace clinician judgment. Outputs must align with clinical guidelines, diagnostic categories, and practitioner expectations to facilitate adoption [26,27]. Alert management is also critical, as excessive notifications can lead to alert fatigue and reduce responsiveness to important warnings [28]. Evidence suggests that clinicians prefer decision-support tools that translate predicted risk into clear, actionable guidance rather than presenting abstract probability scores [28,29,30].

Collectively, these discoveries highlight a key advantage of AI-based modeling over traditional statistical approaches: the ability to identify nonlinear interactions and temporal patterns across heterogeneous data streams. Rather than relying on predefined clinical hypotheses, machine learning models can detect complex multivariate relationships that may signal impending exacerbations before overt clinical deterioration becomes apparent.

### 3.2. Data Sources Used in AI-Based Prediction of COPD Exacerbations

AI-based COPD exacerbation prediction has drawn on multiple data streams, each offering distinct strengths and limitations. Representative studies, data modalities, and selected performance characteristics are summarized in Table 1, Table 2 and Table 3. The narrative below emphasizes how different data sources contribute to prediction, what they reveal about exacerbation risk, and where their limitations constrain clinical translation.

Conceptually, multimodal AI prediction systems operate as layered analytical frameworks that integrate heterogeneous health data streams. Diverse inputs, including electronic health record-derived clinical data, wearable-derived physiological signals, environmental exposure measurements, imaging biomarkers, and patient-reported outcomes, form the data input layer. Machine learning algorithms then analyze these signals to identify temporal trends, cross-domain interactions, and early warning patterns associated with impending exacerbations. The resulting predictive outputs can support risk stratification, remote monitoring, and clinical decision-support strategies aimed at enabling earlier intervention.

#### 3.2.1. Electronic Health Records: Uncovering Healthcare Utilization Patterns

Electronic health records (EHRs) remain among the most widely used and accessible data sources for AI-driven COPD exacerbation prediction. They provide a longitudinal view of patient health across care settings and include both structured data, such as demographics, vital signs, laboratory values, medications, and diagnoses, and unstructured clinical documentation. EHRs are especially well suited to long-term risk stratification and population-level modeling, where historical patterns and cumulative disease burden are central to prediction.

Representative studies show both the scale and practicality of EHR-based modeling. Zeng et al. [14] used longitudinal EHR data from a large healthcare system to develop a prediction framework incorporating demographics, medications, laboratory values, comorbidities, and prior healthcare utilization. Their findings underscored the predictive value of adherence-related prescription features and recent healthcare use, illustrating how EHRs can capture behavioral and utilization patterns beyond diagnostic labels. In a more compact outpatient setting, Liao et al. [13] showed that a smaller set of routinely available EHR variables could still support near-future exacerbation prediction, with prior exacerbation history remaining the most influential predictor (Table 1 and Table 2).

Despite these strengths, EHR-based models face important limitations. Clinical data are irregularly sampled, reflecting episodic healthcare encounters rather than continuous monitoring, which can obscure early physiological deterioration between visits. Missing data are common and often nonrandom, because tests and assessments are ordered selectively. EHRs also provide limited visibility into daily behaviors and environmental exposures that contribute to COPD exacerbation risk.

Despite strong predictive performance in several EHR-based models, important methodological limitations remain. Many studies relied on retrospective single-center datasets, raising concerns about model generalizability across healthcare systems with differing documentation practices and patient demographics. Furthermore, EHR data often contain missing values and inconsistent coding, which may introduce noise into feature engineering and affect model stability when deployed in new clinical environments.

#### 3.2.2. Wearable Devices and Remote Sensors: Detecting Presymptomatic Activity Decline

Wearable devices and in-home sensing technologies extend COPD exacerbation prediction beyond the episodic nature of clinical encounters by providing continuous, objective measurements of behavior, physiology, and environmental exposure. These data streams address a major limitation of EHR-based models by capturing gradual deterioration and short-term fluctuations between visits.

Recent studies suggest that changes in wearable-derived behavioral and physiological signals may occur days before patients report worsening symptoms. Wu et al. [6] showed that declines in objectively measured physical activity preceded symptom-based reporting of exacerbations, highlighting the ability of wearable data to detect subclinical deterioration not readily apparent in routine clinical care. In addition to activity monitoring, wearable and home-based sensing platforms increasingly capture physiological parameters such as respiratory rate, oxygen saturation, heart rate variability, and sleep patterns, which may provide additional early indicators of respiratory instability. Complementary work by Yin et al. [18] demonstrated that respiratory sounds and portable lung function measurements can also support short-term risk estimation, reinforcing the value of home-based physiological monitoring (Table 1 and Table 2).

Overall, wearable and remote sensing studies suggest that continuous monitoring can improve early warning by capturing subtle changes that periodic assessments miss and by reducing reliance on patient recall. Monitoring physiological parameters such as respiratory rate, oxygen saturation, and activity patterns may enable earlier identification of physiological destabilization compared with intermittent clinical assessments. At the same time, sustained adherence, device reliability, data volume, and cost remain important barriers to routine implementation.

Wearable sensor data introduce an inherently temporal dimension that differs from static EHR variables. Consequently, models designed to capture sequential patterns such as recurrent neural networks and LSTM architectures often demonstrate improved performance when applied to activity and physiological monitoring data. These architectures identify gradual behavioral or physiological changes that precede clinically recognized exacerbations.

#### 3.2.3. Imaging Data (CT, Chest Radiography): Identifying Structural Phenotypes Beyond Spirometry

Medical imaging, particularly chest CT, provides structural and functional information about COPD that is not captured by clinical or physiological measures alone. Advances in quantitative image analysis and deep learning have enabled more precise characterization of airway, parenchymal, and extra-pulmonary abnormalities, supporting disease characterization and exacerbation risk assessment.

Several imaging studies demonstrate the value of combining traditional radiologic measurements with data-driven representations. AI-based CT analysis can derive quantitative imaging biomarkers such as airway wall thickness, emphysema burden, regional lung density patterns, and airway branching morphology. These features capture structural heterogeneity in COPD that may not be reflected in conventional spirometric measures. Zhu et al. [16] showed that integrating conventional CT radiomics with deep learning features and epidemiological data improved diagnostic discrimination, suggesting that learned image representations add information beyond handcrafted radiomic measures. Almeida et al. [17] similarly used self-supervised anomaly detection to quantify deviations from normal lung structure, with higher anomaly scores associated with greater disease severity and prior severe exacerbations. Imaging biomarkers have also been used for exacerbation prediction; Wu et al. [19] showed that quantitative CT measures added predictive value beyond conventional clinical indices for forecasting future COPD exacerbations. Although chest CT dominates this literature, Nam et al. [20] suggested that chest radiographs may also provide prognostic information when advanced imaging is unavailable (Table 1 and Table 2).

Imaging studies show that structural biomarkers capture disease heterogeneity not reflected by spirometry. Broader use, however, is constrained by radiation exposure, acquisition variability, computational demands, and the need for standardized pipelines.

Imaging-based models contribute a distinct perspective by capturing structural phenotypes of COPD that are not reflected in spirometry alone. However, their predictive utility for imminent exacerbations remains limited compared with longitudinal physiological or behavioral data. This suggests that imaging features may be more informative for long-term risk stratification rather than short-term exacerbation prediction.

#### 3.2.4. Patient-Reported Outcomes (PRO) and Symptom Data: Capturing Subjective Early Warning Signs

Patient-reported outcomes (PROs) capture symptom burden, functional limitation, and perceived health status directly from the patient’s perspective, providing information only partially reflected in physiological or imaging measures. The growing use of digital health applications has made frequent, longitudinal PRO collection more feasible and has enabled the incorporation of symptom trajectories into exacerbation risk modeling.

Prior studies indicate that changes in self-reported symptoms can precede clinical recognition of exacerbations, supporting the use of PROs for short-term risk assessment and early warning. Chmiel et al. [12] showed that symptom data collected through a digital health application could support near-term exacerbation prediction, while Kor et al. [15] demonstrated that patient-reported respiratory symptoms such as wheezing, dyspnea, and cough were among the strongest contributors to first-time exacerbation risk in an explainable ML framework. Smartphone-based symptom diaries further support real-time tracking, reduce recall bias, and can be integrated with objective streams such as wearable or environmental data (Table 1 and Table 2).

Even so, PRO-based approaches have limitations. Reporting adherence varies across patients, subjective symptom perception introduces inter-patient variability, and symptom changes may lag behind underlying physiological deterioration. As a result, PROs are generally most useful when combined with objective clinical, behavioral, or environmental data rather than used alone.

Patient-reported outcomes provide valuable insight into subjective symptom changes that may precede exacerbations. However, reliance on self-reported data introduces variability related to adherence, recall bias, and reporting consistency. These factors partially explain the variability in predictive performance observed across PRO-based models.

#### 3.2.5. Environmental and Contextual Data: Revealing Individual Pollution Susceptibility

Environmental and contextual factors, including ambient air pollution, meteorological conditions, and circulating respiratory viruses, are established contributors to COPD exacerbations but are largely absent from traditional clinical datasets. Their integration with patient-specific clinical information has emerged as an important frontier in AI-based exacerbation prediction.

Atzeni et al. [7] advanced this area by moving beyond fixed environmental monitoring stations and incorporating personal air quality monitor data. Their study showed that individual-level exposure profiles differed meaningfully from area-level measurements and that cumulative pollutant exposure over several days was associated with exacerbation risk. Evidence from epidemiological and environmental health studies suggests that fine particulate matter (PM_2.5_), nitrogen dioxide (NO_2_), and ground-level ozone (O_3_) are among the pollutants most consistently associated with increased COPD exacerbation risk [7,11].

Importantly, unsupervised clustering identified patient subgroups with different sensitivities to specific pollutants, highlighting heterogeneity that is not apparent in population-level analyses. Jo et al. [11] reached similar conclusions using population-level meteorological data, ambient air pollution measures, and influenza surveillance combined with longitudinal clinical records, demonstrating that environmental and viral indicators contribute materially to daily exacerbation prediction (Table 1 and Table 2).

These studies underscore the importance of incorporating environmental context into exacerbation prediction, especially for short-term and continuous monitoring. Still, challenges remain. Personal monitoring devices are costly and require sustained engagement, while fixed monitoring stations may not reflect individual exposure. Environmental variables are also interdependent, and their effects may vary across regions, limiting transportability.

Environmental exposure models highlight an important shift toward personalized risk prediction. Rather than assuming uniform susceptibility to pollution exposure, AI models identify subgroups of patients whose exacerbation risk is disproportionately influenced by environmental triggers. This capability reflects the strength of machine learning approaches in modeling complex interactions between environmental, behavioral, and physiological variables.

Identification of these environmental sensitivity phenotypes may also support more personalized COPD management strategies, such as targeted environmental exposure counseling, individualized monitoring plans, and preventive interventions during periods of elevated environmental risk.

#### 3.2.6. Multimodal Data Integration: Discovering Cross-Domain Interactions

Growing evidence indicates that integrating multiple data sources yields more robust and clinically informative COPD exacerbation prediction models than reliance on any single modality. Because exacerbations arise from interacting physiological, behavioral, and environmental processes, multimodal approaches are well positioned to capture this complexity.

Representative studies illustrate this advantage. Wu et al. [6] combined wearable-derived activity metrics, home air quality sensor data, and patient-reported symptoms to predict near-term exacerbations, demonstrating that objective lifestyle signals complement symptom-based reporting. Jo et al. [11] integrated longitudinal clinical records with meteorological variables, ambient air pollution, and viral surveillance data, showing the additive value of environmental context. Imaging-based studies support this same principle: Jeon et al. [8] showed that combining spirometry curve images with clinical variables improved exacerbation prediction, while Zhu et al. [16] demonstrated that CT-derived deep learning features complement radiomic and epidemiological data (Table 1 and Table 2).

These studies suggest that exacerbation risk emerges from interactions across domains rather than from any single source. For example, declining activity patterns, increased pollution exposure, and suboptimal medication adherence may together create risk levels that would be missed if each domain were analyzed separately. This shifts COPD risk assessment away from isolated predictors toward a systems-level view of exacerbation vulnerability.

Multimodal integration has been implemented through early fusion of engineered features, late fusion of model outputs, and intermediate fusion of learned latent representations. More recent architectures incorporate attention mechanisms to weight data streams differently across patients and time. Yet multimodal modeling also introduces substantial practical challenges. Data sources differ in temporal resolution, not all patients have access to each modality, and aligning heterogeneous time-stamped data increases preprocessing and computational complexity. As fusion architectures become more sophisticated, model interpretability may decline, reinforcing the need for explainable frameworks and careful clinical validation.

Across these domains, a consistent pattern emerges, models integrating multiple data sources generally outperform those relying on a single modality. EHR-based models capture longitudinal clinical history, wearable sensors detect real-time physiological and behavioral changes, and imaging provides structural phenotyping of disease severity. The complementary strengths of these data streams suggest that future AI systems may achieve the greatest predictive accuracy through multimodal integration rather than reliance on any single data source.

Integrating heterogeneous data sources, including electronic health records, wearable sensor data, imaging biomarkers, patient-reported outcomes, and environmental exposures, allows AI systems to identify cross-domain interactions that may precede COPD exacerbations. Multimodal architectures such as ensemble learning frameworks, hybrid deep learning models, and temporal attention networks increasingly support this systems-level approach to risk prediction.

Figure 2 illustrates a conceptual architecture of an AI-enabled COPD monitoring ecosystem in which diverse clinical, behavioral, imaging, and environmental data streams are integrated to generate predictive risk estimates and clinical decision-support outputs.

### 3.3. Performance of AI Models in Predicting COPD Exacerbations

Reported performance of AI models for COPD exacerbation prediction varies substantially across studies. This heterogeneity reflects differences in data modality, prediction window, outcome definition, cohort composition, and validation strategy. As a result, performance metrics must be interpreted in context rather than compared at face value. Selected study-level metrics are summarized in Table 2, while recurrent methodological limitations affecting interpretation are summarized in Table 3.

#### 3.3.1. Performance Metrics and Benchmarks

Most studies report discrimination metrics, particularly the area under the receiver operating characteristic curve (AUROC), as the primary indicator of model performance. AUROC provides a threshold-independent measure of discriminative ability and facilitates broad comparison across studies, with values above 0.7 and above 0.8 generally considered acceptable and strong, respectively. However, AUROC does not convey false-positive burden, missed events, or clinical usefulness.

Threshold-dependent metrics are therefore also important. Sensitivity is critical in exacerbation prediction because missed events may lead to morbidity, whereas specificity and positive predictive value influence alert burden and clinician trust in real-time or remote monitoring settings. Accuracy is frequently reported but may be misleading in imbalanced datasets where non-exacerbation periods dominate. The F1 score provides a useful summary of the trade-off between false positives and false negatives.

Calibration metrics remain underreported despite their importance for risk communication and decision support. Similarly, only a smaller subset of studies evaluates clinical utility using decision-analytic approaches such as decision curve analysis. Overall, many investigations emphasize discrimination while underreporting calibration and practical utility, a pattern observed across multiple COPD exacerbation prediction studies irrespective of data modality or modeling approach.

#### 3.3.2. Performance Summary Across Studies

Model performance ranges from moderate to high, with the strongest discrimination generally observed in studies that use high-frequency or multimodal data and shorter prediction windows. This pattern is consistent across EHR-based, wearable-based, imaging-based, environmental, and patient-reported data approaches.

Near-term prediction models using continuous monitoring data tend to perform best. Studies using wearable-derived activity measures, environmental sensing, and symptom questionnaires have demonstrated particularly strong performance for 7-day or daily prediction, suggesting that temporally dense and contextually enriched data streams are advantageous for detecting imminent exacerbation [6,7,11] (Table 2).

By contrast, models targeting longer prediction horizons or relying on more episodic data sources tend to show more moderate performance. Symptom-based 3-day models can still support high-sensitivity alerting despite only moderate discrimination, while one-year models based on spirometry curve imaging or comprehensive EHR data provide clinically meaningful but less immediate risk stratification [8,12,14] (Table 2).

Overall, short-term models using behavioral, physiological, and contextual data tend to yield the highest discrimination, whereas longer-term models function more as stratification tools, producing moderate but still useful performance.

#### 3.3.3. Factors Influencing Performance

Several methodological and clinical factors shape model performance. Outcome definition is central: models predicting hospitalization-defined exacerbations often achieve higher specificity but may miss moderate events treated in outpatient settings, whereas broader definitions increase sensitivity at the cost of higher false-positive rates.

Prediction window also matters. Near-term models benefit from dynamic signals such as activity patterns, symptoms, and recent environmental exposures, whereas longer-term models rely more heavily on baseline vulnerability, comorbidities, and prior healthcare use. Data richness is similarly important; models that integrate multiple complementary data sources generally outperform single-modality approaches, although the magnitude of benefit varies.

Cohort characteristics further affect performance. Most models have been developed in patients with moderate-to-severe COPD or frequent exacerbators, while predictive accuracy in milder disease or infrequent exacerbators remains less well characterized. In studies with small sample sizes or non-temporal validation splits, class imbalance and limited event counts may inflate apparent performance. These issues contribute to the wide dispersion of reported metrics and reinforce the need for cautious comparison across studies.

Differences in predictive performance across studies often reflected the alignment between model architecture and the structure of the underlying data. Temporal models, including LSTM and attention-based architectures, demonstrated advantages when applied to longitudinal physiological or symptom data because they can capture sequential dependencies. In contrast, ensemble tree-based methods such as random forests and gradient boosting frequently performed competitively for structured EHR datasets where feature interactions rather than temporal dynamics dominate predictive signal.

The broad range of reported AUROC values (approximately 0.72–0.95 across studies) reflects substantial methodological and dataset heterogeneity. Models developed using larger datasets with richer feature sets, particularly those integrating multimodal inputs such as wearable signals, environmental exposures, and clinical variables, generally demonstrate higher discrimination. In contrast, models based on smaller cohorts, limited feature sets, or episodic clinical data often show more moderate performance. Feature engineering strategies and algorithm selection also contribute to variability; ensemble methods and deep learning architectures may capture complex nonlinear relationships more effectively than simpler models when sufficient data are available. Consequently, differences in dataset size, feature availability, modeling approach, and prediction horizon all contribute to the variability in reported predictive performance across studies.

Common machine learning and deep learning architectures used in COPD exacerbation prediction studies are summarized in Table 4.

#### 3.3.4. Performance Stability and Generalizability

Despite encouraging results, evidence regarding the stability and generalizability of AI-based exacerbation prediction models remains limited. Many published models were developed in single-center cohorts and evaluated only with internal validation. External validation across independent cohorts is still uncommon, and performance may vary with differences in healthcare systems, environmental context, data availability, and exacerbation definitions.

Temporal instability is an additional concern. Shifts in exacerbation patterns, including those observed during the COVID-19 pandemic, highlight the need for ongoing recalibration and monitoring of deployed models. Studies that evaluate performance across time or across settings provide more credible estimates of real-world utility, but such assessments remain the exception rather than the rule. Improving generalizability and temporal robustness is essential for translating research models into sustainable clinical tools.

#### 3.3.5. Clinical Utility Beyond Discrimination

High discriminative performance does not guarantee clinical usefulness. For practical deployment, AI models must support decisions that improve outcomes without generating excessive alert burden or workflow disruption. Several studies have therefore extended evaluation beyond AUROC to include decision-analytic frameworks. For example, Atzeni et al. [7] showed that model-guided intervention could provide net benefit across plausible risk thresholds, whereas Chmiel et al. [12] emphasized the importance of tunable operating points to balance sensitivity and specificity according to clinical priorities.

Many studies focus on predictive discrimination rather than demonstrated clinical impact. While AUROC values provide evidence that exacerbation risk can be predicted with reasonable accuracy, relatively few investigations evaluate whether model-guided interventions improve clinical decision-making, reduce exacerbations, or improve patient-centered outcomes. This gap highlights a central tension in the literature: strong algorithmic performance has not yet translated into consistent evidence of clinical utility.

Overall, the literature suggests that AI models hold strong promise for improving COPD exacerbation prediction, particularly for near-term risk assessment using high-frequency or multimodal data. At the same time, findings across the broader literature continue to highlight persistent challenges related to generalizability, calibration, validation rigor, and demonstration of real-world clinical benefit. Addressing these gaps through standardized evaluation, prospective validation, and careful workflow integration is essential before widespread implementation can be achieved.

### 3.4. Barriers to the Clinical Adoption of AI-Based COPD Exacerbation Prediction

Despite promising predictive performance in research settings, the translation of AI-based COPD exacerbation prediction models into routine clinical practice remains limited. Many of the discoveries highlighted in this review—including early physiologic destabilization detectable through wearable data [19], differential environmental susceptibility phenotypes [7], and spirometry curve morphology patterns beyond conventional indices [8]—depend on data sources and modeling approaches that are difficult to operationalize in everyday care environments. These same innovations introduce practical challenges related to data quality, interpretability, workflow integration, generalizability, and equity [22,38,48].

Representative methodological and implementation barriers are summarized in Table 3. The following sections highlight key categories of obstacles that continue to limit real-world deployment.

#### 3.4.1. Data Quality, Availability, and Integration

The finding that dense behavioral and environmental signals improve short-term exacerbation prediction [7,11,19] highlights a central implementation challenge: clinical information systems rarely capture data with the completeness and synchronization available in research environments. Retrospective models are typically trained on curated datasets with carefully defined variables, whereas operational healthcare data often contain missing values, inconsistent coding, and irregular sampling.

Multimodal models that integrate EHR data with wearables, environmental sensors, imaging, or patient-reported outcomes require accurate temporal alignment across heterogeneous data streams. In practice, this alignment is difficult to maintain across institutions and data systems [29,48]. Although advanced imputation techniques may partially compensate for missing data [37,58], they introduce additional computational complexity and may reduce interpretability. Consequently, the richer and more diverse the input data become, the greater the dependence on robust data infrastructure and preprocessing pipelines.

#### 3.4.2. Model Interpretability and Clinician Trust

Deep learning models often derive predictive value from features that are not part of conventional clinical reasoning. For example, models that analyze the morphology of spirometry curves can detect subtle structural patterns beyond standard spirometric indices [8]. While these features improve discrimination, predictions that rely on unfamiliar representations may reduce clinician trust when they cannot be easily interpreted within existing diagnostic frameworks.

Explainable AI approaches, including SHapley Additive exPlanations (SHAP) and Local Interpretable Model-agnostic Explanations (LIME), have been used to increase transparency by quantifying the contribution of individual features to model predictions [7,15]. These methods can help identify variables that influence predictions and provide insight into model behavior. However, feature-attribution techniques do not establish causal mechanisms and may not fully translate complex model representations into explanations that align with clinical reasoning [25,30]. A ranked list of feature contributions may reveal which variables influenced a prediction but does not necessarily clarify whether the prediction should be trusted or how it should influence clinical decision-making.

In clinical settings, interpretability must support judgment rather than simply describe model mechanics. Clinicians must understand whether the prediction reflects plausible disease processes, whether the signal may represent confounding or data artifacts, and how the prediction should influence patient management. Furthermore, most models report point estimates of risk without explicitly communicating uncertainty, which may further limit clinician confidence in model outputs [48]. As a result, interpretability challenges in COPD prediction extend beyond variable importance to the broader problem of translating algorithmic outputs into explanations that are clinically meaningful, trustworthy, and actionable.

#### 3.4.3. Workflow Integration and Alert Fatigue

AI-based exacerbation prediction aims to detect risk before symptoms become clinically apparent. Evidence that physiological or behavioral changes may occur days before symptom escalation [12,19] creates opportunities for earlier intervention but also introduces workflow challenges. Near-term prediction systems may generate frequent alerts, raising concerns about alert fatigue in already complex clinical environments.

Research in clinical decision support systems demonstrates that excessive or poorly targeted alerts reduce clinician responsiveness and may undermine adoption [28]. Adjustable risk thresholds can help balance sensitivity and specificity, as demonstrated by Chmiel et al. [12], but optimal calibration often depends on the clinical setting. In addition, many models stop at risk prediction without specifying actionable care pathways. Studies of clinician decision support indicate that systems providing guideline-aligned recommendations are more likely to be adopted than those presenting raw risk probabilities alone [29,30]. Without integration into clinical workflows, predictive insights may remain informational rather than actionable.

#### 3.4.4. Generalizability and External Validation

Generalizability remains one of the most persistent challenges in AI-based COPD exacerbation prediction. Several discoveries described in this review—including environmental susceptibility phenotypes [7] and contextual risk modeling approaches [11]—depend on regional exposure patterns, healthcare infrastructure, and patient populations. Models developed in a single institution may therefore perform differently in other healthcare systems with distinct environmental conditions or documentation practices.

Device-dependent data sources introduce additional constraints. Imaging-based models require standardized acquisition protocols, and wearable-based approaches depend on consistent device use and data quality [6,8]. Temporal shifts in disease patterns also affect model stability; for example, changes in exacerbation rates during the COVID-19 pandemic demonstrated how external events can alter predictive relationships [11]. Despite these concerns, rigorous external validation and longitudinal monitoring remain uncommon in the literature, limiting confidence in real-world performance.

#### 3.4.5. Ethical, Equity, and Patient-Centered Considerations

The increasing reliance on multimodal and wearable-driven prediction raises important equity considerations. Although studies using wearable sensors and personal air quality monitors demonstrate technical feasibility [6,7], these technologies may not be equally accessible to all patients. Reliance on smartphones, digital platforms, or home monitoring devices risks excluding individuals with limited technological access or digital literacy.

Many predictive models are also trained on retrospective cohorts from single institutions with limited demographic diversity, and formal fairness analyses across patient subgroups are rarely reported [22,48]. In addition, continuous monitoring and predictive alerts raise questions related to patient autonomy, privacy, and acceptability. Transparent communication about how patient data are collected, analyzed, and used to guide clinical decisions is essential for maintaining trust [29,30,48]. Questions regarding clinical responsibility and liability also remain unresolved when AI predictions influence treatment decisions [29,38].

These barriers highlight an important gap between algorithm development and clinical implementation. While many studies demonstrate promising discrimination metrics, real-world deployment requires addressing challenges related to data integration, workflow compatibility, clinician trust, and external validation. Without these considerations, even highly accurate predictive models may fail to translate into meaningful improvements in patient care.

Taken together, the reviewed studies demonstrate substantial progress in AI-based prediction of COPD exacerbations but also reveal consistent methodological patterns. Predictive performance is strongly influenced by data modality, model architecture, and study design. Temporal modeling approaches show promise for capturing early physiological deterioration, while multimodal integration offers the greatest potential for improving predictive accuracy. At the same time, persistent limitations, including retrospective datasets, limited external validation, and inconsistent outcome definitions, continue to constrain the translation of these models into routine clinical practice.

## 4. Discussion

Artificial intelligence-based COPD exacerbation research has progressed beyond proof-of-concept prediction toward revealing deeper structural and temporal patterns in disease destabilization. Three major discoveries emerge across diverse modeling strategies and data sources: (1) exacerbations are preceded by measurable physiologic and behavioral deterioration several days before symptom recognition; (2) environmental susceptibility and medication adherence interact across patient subgroups; and (3) deep learning analysis of full spirometric and imaging data identifies structural phenotypes not captured by traditional FEV_1_-based staging.

These discoveries shift the conceptual framing of COPD exacerbation risk from static severity classification to dynamic, multidimensional vulnerability.

At the same time, the evidence reviewed in this manuscript highlights a persistent translational gap. Many studies demonstrate promising predictive discrimination, yet comparatively few evaluate whether model-guided predictions improve clinical decision-making or patient outcomes. This distinction between algorithmic accuracy and clinical utility represents a central challenge for translating AI-based exacerbation prediction into routine care.

### 4.1. Principal Discoveries and Their Implications

Three consistent discoveries emerge from the studies synthesized in this review. First, exacerbations are preceded by measurable physiologic and behavioral deterioration several days before symptom recognition. Second, environmental exposures and medication adherence interact across patient subgroups, revealing differential susceptibility to exacerbation triggers. Third, deep learning analysis of complete spirometry curves and imaging data identifies structural phenotypes not captured by conventional FEV_1_-based staging.

Unlike conventional spirometric indices such as FEV_1_, which summarize airflow using a single value, full-curve analysis preserves the detailed morphology of the flow–volume and volume–time curves across the entire expiratory maneuver. Deep neural networks can analyze subtle variations in curve shape, including early and late expiratory flow dynamics, inflection points, and curvature patterns that reflect heterogeneous airway obstruction and small-airway dysfunction. By learning spatial and temporal relationships across the entire spirometric waveform, these models can detect physiologic abnormalities that may not be captured by summary indices alone.

Together, these findings shift the conceptual model of COPD exacerbation risk from static severity classification toward dynamic, multidimensional vulnerability. Rather than representing abrupt and unpredictable events, exacerbations appear to develop through progressive destabilization detectable through changes in activity patterns, environmental exposure, and physiological signals. This perspective aligns COPD management with precision medicine approaches by emphasizing individualized risk assessment and anticipatory intervention rather than reactive crisis management.

### 4.2. From Discovery to Clinical Translation

If exacerbations begin 7–14 days before hospitalization, the clinical question becomes how to operationalize that window. Potential interventions include the use of temporary bronchodilator intensification, early corticosteroid initiation under remote supervision, short-course antibiotics when infection signatures are detected, enhanced telemonitoring, or targeted environmental exposure mitigation. The practical value of prediction lies in enabling timely stabilization.

The recognition of differential environmental susceptibility demonstrates that exposure guidelines could evolve toward individualized thresholds. The integration of real-time environmental monitoring with patient-specific risk modeling may enable adaptive pharmacologic adjustment or proactive advisories during high-risk periods.

Translation depends on interpretability, workflow integration, alert calibration, and clinician trust. Without these, the models risk contributing to alert fatigue rather than clinical benefit.

### 4.3. Limitations

#### 4.3.1. Limitations of the Current Evidence Base

Despite promising predictive performance, most studies remain retrospective and single-center, limiting generalizability. External validation is inconsistent, and model performance often declines when applied to new populations. Because AI models identify statistical associations rather than causal mechanisms, uncertainty remains regarding which factors represent modifiable drivers versus risk correlates. In addition, heterogeneity in outcome definitions and reporting complicates cross-study comparisons. These limitations highlight the need for prospective validation, standardized exacerbation definitions, and cautious interpretation of discrimination metrics.

Another important limitation in the current evidence base involves potential bias arising from dataset composition. Many AI models for COPD exacerbation prediction are trained using data derived from single health systems or geographically limited populations, which may not reflect the demographic and socioeconomic diversity of the broader COPD population. In addition, exacerbation events are relatively infrequent compared with stable disease periods, creating class imbalance that can influence model training and inflate apparent predictive performance if not carefully addressed. Such imbalance may bias models toward predicting the majority class and reduce sensitivity for clinically meaningful exacerbation events. Demographic disparities in training datasets may further limit model generalizability across age groups, ethnic backgrounds, and healthcare settings. Addressing these challenges will require more diverse datasets, transparent reporting of cohort characteristics, and external validation across multiple populations before AI-based COPD prediction tools can be reliably implemented in clinical practice.

#### 4.3.2. Limitations of the Review Methodology

This review also has methodological limitations. Although a structured search across multiple databases was performed, the study was conducted as a narrative review rather than a formal systematic review or meta-analysis. Consequently, some risk of selection bias remains, and relevant studies may not have been captured. The goal was therefore not to provide an exhaustive inventory of all published models but to synthesize representative studies illustrating the evolving methodological landscape of AI-driven COPD exacerbation prediction.

### 4.4. Future Research Priorities

Translating AI-based COPD exacerbation prediction from research prototypes into clinically useful tools will require advances in methodology, data infrastructure, implementation science, and equitable deployment. Future research should build on the discoveries described in this review while prioritizing real-world clinical impact.

Prospective validation represents a critical next step before AI-based COPD exacerbation prediction models can be integrated into routine clinical care. Most currently published models have been evaluated using retrospective datasets, which may overestimate performance due to dataset-specific patterns and incomplete representation of real-world clinical variability. Future research should therefore prioritize prospective cohort studies and randomized clinical trials that evaluate whether AI-guided prediction systems improve patient outcomes compared with standard care.

Such studies should assess clinically meaningful endpoints including exacerbation frequency, hospitalization rates, healthcare utilization, quality of life, and mortality. Pragmatic trials embedded within routine clinical workflows may be particularly valuable for evaluating real-world effectiveness, clinician adoption, and unintended consequences such as alert fatigue. Only through rigorous prospective validation across diverse healthcare settings can AI-based COPD prediction systems demonstrate the reliability, safety, and clinical benefit required for widespread clinical implementation.

#### 4.4.1. Methodological Innovations

Most current studies rely on retrospective datasets, which limits evidence of real-world effectiveness. Prospective evaluation is therefore essential. Randomized or pragmatic trials comparing AI-guided care with standard management should prioritize patient-centered outcomes such as exacerbation frequency, hospitalization rates, quality of life, and healthcare utilization rather than predictive accuracy alone [29,37,38].

Future research should test whether AI-derived discoveries translate into effective interventions. For example, studies should evaluate whether reducing PM_2.5_ exposure in environmentally sensitive phenotypes [7], initiating treatment during the 7–14-day physiologic decline window detected through wearable monitoring [6], or targeting therapies based on imaging-derived phenotypes [16,42] improves clinical outcomes. Multicenter validation and continuous model monitoring will also be necessary as care environments and exposures evolve [9,38,48].

#### 4.4.2. Data Infrastructure and Standards

Progress is constrained by fragmented exacerbation definitions and inconsistent data infrastructure. Standardized definitions, such as those proposed in the Rome Proposal, would improve comparability across studies [9,21]. Interoperability standards including Health Level 7 Fast Healthcare Interoperability Resources (HL7 FHIR) may support model portability across healthcare systems [25,36], while federated learning offers a privacy-preserving approach to multi-site collaboration [31,32,33,34].

#### 4.4.3. Clinical Implementation

Successful deployment will require human-centered design and integration into clinical workflows. AI-based monitoring systems intended for clinical decision support may also require regulatory oversight as software-based medical devices, with potential approval pathways including the U.S. Food and Drug Administration (FDA) 510(k) or De Novo processes and CE marking under the European Medical Device Regulation (MDR). However, the regulatory landscape for adaptive AI and machine learning systems continues to evolve as these technologies become more widely used in healthcare.

Beyond regulatory approval, predictions must be embedded within clinical workflows that clinicians already use, such as electronic health record dashboards, mobile alerts, or patient-facing monitoring platforms. Poorly integrated systems may generate predictions without influencing clinical decisions. Building on these principles, emerging clinical validation studies further reinforce the real-world applicability of AI-driven approaches. Machine learning models developed using routinely collected clinical data have demonstrated strong predictive performance for clinically meaningful outcomes such as hospitalization, readmission, and disease progression, supporting their role in risk stratification and clinical decision support [14,41]. In parallel, digital health studies utilizing sensor-enabled inhalers and wearable monitoring systems have demonstrated the ability to detect early physiological changes and predict exacerbations several days in advance, highlighting the feasibility of integrating continuous monitoring into routine care pathways [6,35,36]. Collectively, these findings underscore both the clinical relevance of AI-based predictions and the growing need for regulatory and implementation frameworks that ensure safe, interpretable, and actionable deployment in practice.

Careful calibration of alert thresholds and communication of model uncertainty will also be essential to minimize alert fatigue and maintain clinician trust [12,28,30,48,59,60]. Implementation science frameworks such as RE-AIM and CFIR remain underused in AI-based COPD research but offer structured approaches for evaluating adoption and sustainability [61,62].

#### 4.4.4. Equity and Precision Medicine

AI-enabled monitoring systems introduce important equity considerations. Wearable-based early warning systems [6] and environmental monitoring [7] may disproportionately benefit patients with greater technological access. Ensuring equitable implementation will require inclusive design, subgroup evaluation, and monitoring for algorithmic bias [29,30,48]. Emerging technologies, including transfer learning [45], edge computing [6,51], and digital twin models [38], may further support precision medicine approaches that tailor prevention strategies to each patient’s biological, behavioral, and environmental context.

Advancing these priorities will be essential for translating AI-based COPD exacerbation prediction from promising analytic capability into clinically actionable prevention strategies.

## 5. Conclusions

This narrative review highlights three emerging discoveries from AI-driven COPD exacerbation research: identification of preclinical physiological deterioration windows preceding symptom recognition, recognition of patient phenotypes with differential environmental and behavioral sensitivity, and detection of multidomain risk interactions and structural phenotypes not captured by traditional spirometric indices. Across diverse data sources and modeling strategies, predictive performance ranges from moderate to strong, with the highest discrimination observed in short-term models integrating multimodal inputs such as electronic health records, wearable-derived activity data, environmental exposures, imaging biomarkers, and patient-reported outcomes.

Collectively, these findings challenge the prevailing reactive paradigm of COPD management. Evidence that exacerbations develop gradually over several days suggests opportunities for earlier intervention, while phenotype-specific environmental and behavioral sensitivities support more individualized prevention strategies.

Despite these advances, translation into routine clinical care remains limited. Many models rely on retrospective datasets, lack external validation, and have not yet demonstrated improvements in patient-centered outcomes.

Future research must therefore move beyond predictive accuracy toward prospective validation, real-world deployment studies, and integration with telemedicine and remote monitoring platforms. Embedding AI-generated risk predictions within digital health ecosystems, including wearable monitoring systems, telehealth platforms, and electronic health record dashboards, may enable earlier clinical intervention and more personalized disease management.

Ultimately, the value of AI in COPD care will be determined not by how accurately it predicts exacerbations, but by whether it enables earlier intervention and meaningful prevention of disease destabilization.

## Figures and Tables

**Table 1:** Representative Studies and Data Sources Used in AI-Based COPD Exacerbation Prediction.

Study	Data Source	Modeling Approach	Key Inputs	Prediction Window	Key Insight
Wu et al. [6]	Wearable sensors, environmental monitors, and symptom questionnaires	Machine learning models	Activity patterns, environmental exposure, symptom reports	7 days	Demonstrated that declines in physical activity precede symptom-reported exacerbations
Atzeni et al. [7]	Personal air quality monitors	Unsupervised clustering and predictive modeling	Individual pollutant exposure profiles	Short-term prediction	Identified environmental susceptibility phenotypes and heterogeneity in pollution sensitivity
Jo et al. [11]	Clinical records and environmental datasets	Gradient boosting models	Meteorology, air pollution, influenza surveillance, clinical history	Daily prediction	Achieved strong predictive performance integrating environmental context
Chmiel et al. [12]	Patient-reported symptoms via digital health platform	Machine learning classification	Symptom trajectories	3 days	Demonstrated feasibility of symptom-based early warning systems
Liao et al. [13]	Electronic health records	Supervised ML models	Demographics, prior exacerbations, clinical variables	Near-term prediction	Showed that compact EHR feature sets can support practical clinical prediction
Zeng et al. [14]	Longitudinal EHR datasets	ML prediction framework	Demographics, medications, lab values, healthcare utilization	1 year	Demonstrated value of large-scale EHR data for long-term risk prediction
Kor et al. [15]	EHR and symptom variables	Explainable ML with SHAP	CAT scores, respiratory symptoms	Exacerbation prediction	Demonstrated transparent risk explanations using SHAP
Zhu et al. [16]	CT imaging and clinical data	Variational autoencoder + radiomics	Deep imaging features, epidemiologic variables	Disease characterization	Showed complementary value of learned imaging features
Almeida et al. [17]	CT imaging	Self-supervised anomaly detection	Structural lung deviations	Severity prediction	Demonstrated unsupervised imaging

**Table 2:** Performance Metrics Reported Across Representative COPD Exacerbation Prediction Studies.

Study	Data Source	Prediction Window	Model Type	Reported Performance
Wu et al. [6]	Wearables, environment, and symptoms	7 days	Machine learning models	Strong discrimination for near-term exacerbation prediction
Jo et al. [11]	Clinical, environmental, and surveillance	Daily	Gradient boosting	AUROC = 0.935
Atzeni et al. [7]	Personal environmental monitoring	Short-term	ML models	High discrimination with subgroup stratification
Chmiel et al. [12]	Patient-reported symptom data	3 days	ML classification	AUROC = 0.727
Jeon et al. [8]	Spirometry curve imaging and clinical variables	1 year	Deep learning + clinical features	Moderate discrimination
Zeng et al. [14]	Large-scale EHR data	1 year	ML prediction models	AUROC = 0.866

**Table 3:** Common Methodological and Implementation Barriers in AI-Based COPD Exacerbation Prediction.

Barrier Category	Description	Supporting References
Data Heterogeneity	Substantial variation in patient populations, feature definitions, data sources (e.g., EHRs, wearables), and outcome measures limits comparability across studies and complicates robust model development.	[9,13,14,18]
Limited Generalizability	Many models are developed using single-center or highly selected cohorts, reducing external validity across healthcare systems, geographic regions, and diverse patient populations.	[13,14,19]
Small Sample Sizes	Limited cohort sizes in several studies restrict the ability to capture variability in exacerbation patterns and reduce statistical robustness and model stability.	[9,18]
Retrospective Study Design	Heavy reliance on retrospective datasets introduces selection bias, limits causal inference, and reduces prospective clinical applicability.	[9,18,20]
Inconsistent Outcome Definitions	Variability in defining exacerbations (e.g., symptom-based vs. healthcare utilization-based) affects model training, evaluation, and comparability across studies.	[18,21]
Missing and Incomplete Data	EHR and clinical datasets frequently contain missing, sparse, or irregularly sampled data, which can bias model outputs and reduce reliability.	[14,22]
Overfitting and Internal Validation Bias	Many studies rely on internal validation without sufficient external testing, increasing the risk of overfitting and inflated performance metrics.	[9,18,20]
Lack of External Validation	Few models are validated across independent datasets or healthcare systems, limiting confidence in generalizability and real-world performance.	[9,18]
Limited Interpretability (Black-Box Models)	Complex machine learning and deep learning models often lack transparency, reducing clinician trust and hindering adoption in clinical practice.	[23,24,25]
Integration into Clinical Workflows	AI systems frequently lack alignment with clinical decision-making processes, electronic health record systems, and care pathways, limiting usability and adoption.	[26,27]
Alert Fatigue and Usability Challenges	Poorly calibrated alerts and excessive notifications can overwhelm clinicians, contributing to alert fatigue and reduced responsiveness to critical warnings.	[28]
Actionability of Outputs	Models that provide risk scores without clear clinical recommendations or decision support are less useful than systems offering actionable guidance.	[28,29,30]
Data Privacy and Ethical Concerns	Regulatory, privacy, and data governance challenges limit data sharing, multi-institutional collaboration, and large-scale model development.	[31,32,33,34]
Wearable and Sensor Limitations	Issues such as signal noise, patient adherence, device variability, and cost affect the reliability and scalability of real-world monitoring systems.	[35,36]

**Table 4:** Machine Learning and Deep Learning Models commonly used in COPD exacerbation prediction studies.

Model Architecture Used in COPD Prediction Studies	General Type of Method	Typical Data Modalities Used	Key Strengths for COPD Exacerbation Prediction	Representative Studies
Random Forest	Ensemble tree-based machine learning	Electronic health records (EHR), clinical variables, laboratory results	Captures nonlinear feature interactions, robust to noisy clinical data, performs well with structured tabular datasets	[13,14,48]
Gradient Boosting (e.g., XGBoost, LightGBM)	Ensemble boosting machine learning	EHR data, environmental exposures, epidemiologic variables	Strong predictive performance with structured datasets, handles heterogeneous clinical variables effectively	[11,14]
Support Vector Machine (SVM)	Kernel-based supervised machine learning	Clinical variables, physiological measurements	Effective in moderate-sized datasets and high-dimensional feature spaces	[37,48]
Convolutional Neural Networks (CNN)	Deep learning architecture	Imaging data (CT scans, chest radiographs), spirometry curve representations	Extracts complex spatial patterns from imaging or signal data not captured by traditional features	[8,16,20]
Recurrent Neural Networks (RNN) and Long Short-Term Memory (LSTM) Networks	Temporal deep learning architecture	Wearable sensor streams, symptom trajectories, longitudinal physiological signals	Captures sequential temporal patterns and dynamic physiological changes preceding exacerbations	[6,19]
Hybrid Models (e.g., CNN–LSTM or Multimodal Fusion Architectures)	Combined deep learning approaches	Multimodal data including imaging, wearable signals, environmental data, and clinical variables	Integrates heterogeneous data sources to capture multidomain risk interactions	[6,16,48]
Explainable Machine Learning Models (e.g., models with SHAP interpretation)	Interpretable machine learning frameworks	EHR data, symptom variables, environmental exposures	Improves transparency of feature importance and supports clinician understanding of risk predictions	[7,15]

## Data Availability

No new data were created or analyzed in this study.

## Abbreviations

The following abbreviations are used in this manuscript:

AI	Artificial Intelligence
AUC	Area Under the Curve
AUROC	Area Under the Receiver Operating Characteristic Curve
CFIR	Consolidated Framework for Implementation Research
CNN	Convolutional Neural Network
COPD	Chronic Obstructive Pulmonary Disease
CT	Computed Tomography
DL	Deep Learning
EHR	Electronic Health Record
FEV_1_	Forced Expiratory Volume in one second
GNN	Graph Neural Networks
HL7 FHIR	Health Level Seven–Fast Healthcare Interoperability Resources
LIME	Local Interpretable Model-Agnostic Explanations
LSTM	Long Short-Term Memory Network
ML	Machine Learning
MMI	Multimodal Integration
NO_2_	Nitrogen Dioxide
PM_2.5_	Particulate matter with a diameter of 2.5 μm or smaller
PRO	Patient Reported Outcomes
RE-AIM	Reach, Effectiveness, Adoption, Implementation, and Maintenance
RF	Random Forest
SHAP	SHapley Additive exPlanations
SVM	Support Vector Machine
VAE	Variational Autoencoders
XAI	Explainable Artificial Intelligence
